# Development of a synoptic MRI report for primary rectal cancer

**DOI:** 10.1186/1748-5908-4-79

**Published:** 2009-12-02

**Authors:** Gillian Spiegle, Marisa Leon-Carlyle, Selina Schmocker, Mark Fruitman, Laurent Milot, Anna R Gagliardi, Andy J Smith, Robin S McLeod, Erin D Kennedy

**Affiliations:** 1 Department of Surgery, Toronto General Hospital, Toronto, ON, Canada; 2 Department of Radiology, St. Joseph's Health Centre, Toronto, ON, Canada; 3 Department of Surgery, Sunnybrook Health Sciences Centre, Toronto, ON, Canada; 4 Department of Health Policy, Management and Evaluation, University of Toronto, Toronto, ON, Canada; 5 Department of Surgery, Mount Sinai Hospital, Toronto, ON, Canada

## Abstract

**Background:**

Although magnetic resonance imaging (MRI) is an important imaging modality for pre-operative staging and surgical planning of rectal cancer, to date there has been little investigation on the completeness and overall quality of MRI reports. This is important because optimal patient care depends on the quality of the MRI report and clear communication of these reports to treating physicians. Previous work has shown that the use of synoptic pathology reports improves the quality of pathology reports and communication between physicians.

**Methods:**

The aims of this project are to develop a synoptic MRI report for rectal cancer and determine the enablers and barriers toward the implementation of a synoptic MRI report for rectal cancer in the clinical setting. A three-step Delphi process with an expert panel will extract the key criteria for the MRI report to guide pre-operative chemoradiation and surgical planning following a review of the literature, and a synoptic template will be developed. Furthermore, standardized qualitative research methods will be used to conduct interviews with radiologists to determine the enablers and barriers to the implementation and sustainability of the synoptic MRI report in the clinic setting.

**Conclusion:**

Synoptic MRI reports for rectal cancer are currently not used in North America and may improve the overall quality of MRI report and communication between physicians. This may, in turn, lead to improved patient care and outcomes for rectal cancer patients.

## Background

Colorectal cancer is the third leading cause of death from cancer worldwide. There are over 639 000 deaths annually from rectal cancer [1]. The two main goals of rectal cancer treatment are to cure cancer and prevent local recurrence. Both pre-operative chemoradiation and surgical technique have been shown to influence the rate of local recurrence, which is a quality indicator for the treatment of rectal cancer [2-5].

In North America, guidelines recommending pre-operative chemoradiation for patients with Stage II and Stage III rectal cancer have been published, because this has been shown to decrease the risk of local recurrence and has fewer side effects than post-operative chemoradiation [3,4,6]. Therefore, accurate staging of rectal cancer at the time of diagnosis is essential in order to assess the need for pre-operative chemoradiation.

Total mesorectal excision (TME) is a surgical technique in which the rectum and surrounding lymph nodes are removed *en bloc*. TME is necessary in order to achieve a negative circumferential margin, which has also been shown to decrease the risk of local recurrence [3]. Thus, diagnostic imaging is critical for pre-operative planning to determine whether a negative circumferential margin can be achieved and the extent of surgery that will be required to achieve this negative margin [7].

To date, magnetic resonance imaging (MRI) is widely available and an accurate imaging modality for rectal cancer staging and pre-operative planning [7-9]. Despite this, there has been little systematic investigation into how the MRI results are interpreted or reported by clinicians [10]. This is an extremely important area of research, because optimal patient care and clinical outcomes (*i.e.*, risk of local recurrence) require accurate interpretation and documentation of the MRI; as well as clear communication of this information to members of the multidisciplinary team, which include: surgeons, radiation oncologists, medical oncologists, and pathologists.

The use of a clinical synoptic report can facilitate communication between the members of the multidisciplinary cancer care team [11,12]. Synoptic means 'summarized' and refers to the presentation of information in a tabular, rather than descriptive form. Templates are created specifically for a particular setting and can be filled in by the reporting physician. Synoptic reports are of great value because they ensure that all of the information required to guide treatment is addressed and included in the report [11,12]. Synoptic reports not only help to ensure completeness, but also consistency in reporting. In addition, the synoptic format facilitates efficient extraction of information for members of the multidisciplinary team and for registry, data collection, and research purposes. Previous studies have shown that pathologic synoptic reports result in more complete reports for patients with breast and colorectal cancer, and that clinicians find it easier to interpret clinically pertinent information from them [13,14]. Currently, in Ontario, pathologic synoptic reports for cancer have been implemented across the province, and a recent report from Cancer Care Ontario (CCO) shows that synoptic pathology reports are more complete than non-synoptic pathologic reports [15]. Despite the benefits of synoptic clinical reports, to date there has been no synoptic MRI report developed or implemented for rectal cancer in North America [16].

### Aims

The specific aims of this project are to develop a synoptic MRI report for primary rectal cancer, and to elicit the opinions of radiologists regarding enablers and barriers towards the implementation and sustainability of synoptic reports in clinical practice.

## Methods and design

Prior to the start of the project, ethics approval will be obtained.

### Specific aim one: To develop a synoptic MRI report for primary rectal cancer

### Overview

**Figure 1:**
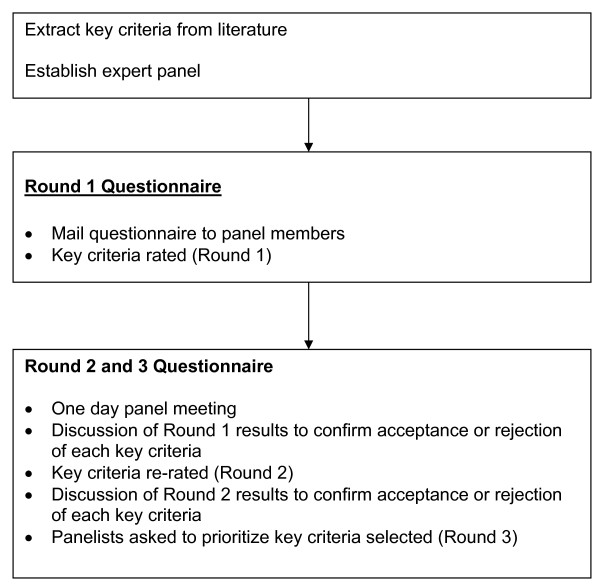
**Process used to select and prioritize key criteria for synoptic MRI report**. This outline will serve as a template for our study to establish what items are essential for the MRI synoptic report and order them by importance.

A three-step Delphi process involving an expert panel will extract the key criteria for an MRI report to guide pre-operative chemoradiation and surgical planning [5,17]. The Delphi approach uses questionnaires to elicit anonymous responses over a number of rounds with controlled feedback; the modified Delphi process involves an in-person meeting of participants. For this study, the expert panel will rate and select key criteria in two consecutive rounds (round one and two) of questionnaires. During round three, the panel will prioritize the key criteria selected from the previous two rounds. Round one will be conducted as a mailed questionnaire and Round two and Round three will involve a one-day panel meeting (Figure 1).

### Panel selection

Hospital Chief Executive Officers and Regional Vice Presidents of Cancer Services from community and tertiary care hospitals in Ontario, Canada will be asked to nominate practicing clinicians that provide care to rectal cancer patients and have demonstrated clinical leadership through research or administrative responsibilities to serve as panel members. The population of Ontario is approximately 13 million, and all health care services are publicly funded by the government. The goal will be to assemble a 15-member multidisciplinary panel representative of practicing clinicians in Ontario. The panel will consist of surgeons (n = 4), radiation oncologists (n = 3), medical oncologists (n = 2), radiologists (n = 4), and pathologists (n = 2) who care for rectal cancer patients in Ontario and involve representation from both academic and community hospitals from different Local Health Integration Networks (LHINs) across Ontario. For this particular panel, we will specifically seek pathologists that are using the synoptic pathology report at their centre, because these individuals will have significant insight into the enablers and barriers for implementation and sustainability of synoptic reports. Nominated clinicians will be contacted by mail to describe the intended process, expected time commitment, and confirm their interest in being involved. It is expected that we will need to contact approximately 45 nominated clinicians to achieve the final 15-member panel (expected participation rate approximately 30%). In order to improve physician participation on the panel, a $500 honorarium will be offered and travel expenses to the one-day meeting will be reimbursed.

### Data collection and analysis

#### Literature search

A literature search will be conducted in MEDLINE using indexing and keywords to identify key criteria on MRI that are important for guiding treatment with respect to pre-operative chemoradiation and pre-operative surgical planning. This literature search will be augmented by an Internet search for 'gray literature' such as government reports. Articles will be included in this review if they were published in the English language from 1990 to present and describe key elements or templates for MRI reporting of rectal cancer. Data on type of article, citation, and key criteria will be extracted and tabulated to generate an evidence table. A preliminary literature search yielded the key criteria shown in Additional file 1.

#### Round one

The key criteria retrieved during the literature search will be formatted as a questionnaire and distributed by regular mail along with the evidence table and a stamped, addressed return envelope. Respondents will be asked to rate the importance of each key criteria to guide treatment on a seven-point scale (one = disagree and seven = agree), provide written comments, and suggest additional indicators not included in the questionnaire that warrant consideration by the panel. A reminder e-mail will be sent two weeks from the initial distribution, and non-responders will also be contacted by telephone to promote return of all questionnaires.

Questionnaire responses will be entered into Microsoft Excel, and frequencies will be calculated and a summary report will be prepared. The report will be organized according to key criteria that achieved: strong consensus for acceptance (eight or more panel members agreed that the item was a key criteria by selecting five, six, or seven on the scale); strong consensus for exclusion (eight or more panel members agreed the item was not a key criteria by selecting one, two, three or four on the scale); unclear consensus (seven panel members agreed the item was a key criteria by selecting five, six, or seven on the scale, and seven or more panel members agreed the item was not a key criteria by selecting one, two, three or four on the scale); and newly suggested key criteria [17].

The summary report will be distributed back to the panel members who will reconvene at a one-day meeting. Acceptance, rejection, or the need for further consideration of each key criterion will be reviewed and confirmed through discussion at the one-day meeting at the start of round two [17]

#### Round two

Following this discussion, key criteria still lacking consensus from round one will be formatted into a round two questionnaire similar in format to round one. The round two questionnaire will include the frequency distribution of the round one responses and a list of previously submitted comments. The round two questionnaire will be distributed to the panel members along with their completed round one questionnaire for reference. Panel members will be asked to rate the round two key criteria. Responses will be summarized as before, then distributed to the panel members who will discuss the round two criteria and confirm their acceptance or rejection of each key criteria [17].

#### Round three

Next, all key criteria selected from round one and two will be included in a third and final questionnaire. Panel members will be asked to prioritize the key criteria by choosing the items they perceive to be the most important to guide treatment in terms of need for pre-operative chemoradiation and surgical planning.

#### Synoptic report

The final product from this process will be a prioritized list of key criteria for the MRI report necessary to guide treatment with respect to pre-operative chemoradiation and surgical planning. These prioritized key criteria will be used to develop a synoptic MRI template. The MRI synoptic template will be circulated to the expert panel to review content and format. A teleconference will be arranged with the expert panel for final comments and suggestions regarding the final format of the MRI synoptic report. The project team will meet following this teleconference to discuss these final comments and suggestions, make modifications as necessary, and finalize the synoptic MRI report. The final synoptic MRI report will be robust because it will have been developed through an extensive review of the literature and rigorous consensus process with an expert panel representative of clinicians.

### Specific aim two: To elicit the opinions of radiologists regarding enablers and barriers towards the implementation and sustainability of synoptic reports in clinical practice

### Overview

Specific aim two will act as a needs assessment to investigate radiologists' attitudes towards synoptic clinical reports and enablers and barriers to the use of these reports in clinical practice. No existing models describe implementation of synoptic clinical reports, or factors that can influence their use and associated outcomes. A model of clinical guideline compliance supports that there are sequential, cognitive, and behavioural steps physicians make as they comply with clinical guidelines [18]. These sequential steps are awareness, agreement, adoption, and adherence. The significance of this model is that it provides those interested in guideline adherence a more detailed understanding of what occurs when physician care deviates from guidelines and assists in developing more effective strategies to overcome these obstacles [18]. This model is germane to this project, as physician adherence, in particular radiologists, will be critical for the successful implementation of the synoptic MRI report for rectal cancer. It will also allow for exploration of other potential organizational or system barriers that influence physician behaviour. Therefore, we will use the model developed by Cabana et. al. as the conceptual framework for this project (Additional file 2) [18,19]. This conceptual framework will serve as a guide for aim two in which radiologists will be interviewed to elicit their opinions about clinical synoptic reports and enablers and barriers to their use in clinical practice. This information will be critical in order to develop effective strategies for implementation of the synoptic MRI report (specific aim one) for primary rectal cancer.

### Physician interviews

Interviews will be conducted by telephone with 20 Radiology Department Heads and 20 radiologists across Ontario, for a total of 40 interviews. These individuals will be selected in non-mutually exclusive fashion by age (<50 years, >50 years), gender (male, female), geographic location (Ontario, LHINs) and type of hospital (academic, community). These details are available from the Ontario College of Physicians and Surgeons (CPSO) internet site, which is a publicly accessible listing of all active physicians in Ontario and is updated annually. Radiologists on the expert panel (specific aim one) will not be eligible for participation in the interviews for specific aim two.

Eligible participants will be contacted by mail with an interview invitation and consent form. A reminder will be mailed to non-responders two weeks after the initial mail out, followed by a telephone call to the remaining non-responders two weeks after the second mail out.

To encourage participation, strategies to increase survey response rates include a hand signed, personalized cover letter on institutional letterhead and a pre-addressed, stamped return envelope will be used [20,21]. In addition, an honorarium of $100 will be given to each participant for their time commitment. It is expected that 150 invitations will need to be mailed in order to conduct 40 interviews assuming a participation rate of approximately 30%.

### Data collection

Semi-structured interviews will be conducted by telephone and all interviews will be audio-recorded and later transcribed by an external professional. The main objectives of the interviews are: to explore participants opinions of, and current experience with, clinical synoptic reports; to explore participants perceptions of enablers and barriers to the use and sustainability of clinical synoptic reports; and to provide any suggestions or recommendations for implementation and sustainability of the synoptic MRI report (or synoptic pathology report) at their centre. Prior to the start of the study, the interviews will be pilot tested on a small number of physicians to refine wording and flow of questions.

### Qualitative research methods and data analysis

Standard principles of qualitative research will be used to sample the participants representing various characteristics, contexts, and settings [22]. Hence, sampling will be purposive to select individuals whose opinions may vary according to these attributes. In qualitative research, detailed information from a representative rather than a large number of cases is needed. Sample size is capped when no further unique themes emerge from successive interviews (informational redundancy) [22]. This is determined at the time of the data analysis, which is conducted concurrently with the data collection. If informational redundancy is not achieved, additional interviews will be conducted.

An inductive, grounded approach will be used for qualitative analysis of interview transcripts using constant comparative analysis [22-24]. This means that themes will be allowed to emerge from the collected data, and progress through three defined processes: description, categorical/conceptual ordering, and theorizing [22,23,25]. This involves repeated reading of transcripts, development of a coding scheme reflecting unique ideas, application of the coding scheme to transcript text, and grouping of coded text by theme. Consistent with constant comparative analysis, open and axial coding of interview transcripts will occur simultaneously because data collection and analysis are concurrent [23] Open coding recognizes ideas or concepts identified by study participants by analyzing transcripts line-by-line in their entirety, and groups concepts together to form categories and subcategories, often using participants' own words as code names to ensure groundedness [23]. In this initial stage of constant comparative analysis, data is coded in every way possible to uncover all ideas.

Next, axial coding will be used to make connections between categories and subcategories of codes. Codes generated from open coding will be collapsed and grouped into mutually exclusive categories focusing on three interrelated aspects of Strauss and Corbin's (1990) coding paradigm: individual actions or behaviours, situational context, and consequences of the behaviours [22]. Repeating ideas will be assembled into themes based on content similarity. A theme is an implicit topic that organizes a group of repeating ideas. Themes will be similarly reviewed and assembled into abstract theoretical constructs based on their relation to one another and their ability to explain factors influencing the implementation of clinical synoptic reports. Theoretical constructs organize themes into larger, more abstract ideas. Themes and theoretical constructs will be tabulated to compare physician opinions and enablers and barriers of implementation of clinical synoptic reports by physician, as well as contextual factors. Finally, theoretical constructs will be organized into a theoretical narrative that summarizes what was learned and bridges the research objectives with participants' subjective experience.

To improve the reliability of these findings, two investigators will individually analyze and code all transcripts. They will meet to compare findings and achieve consensus through discussion. Collaborative coding by multiple individuals minimizes the chance that important thematic ideas are overlooked, and ensures that the organization of the data and the resulting conceptual theory is transparent [25].

Specific aim two will contribute two important deliverables. First, it will provide a framework to describe the implementation of clinical synoptic reporting that can be used for the purposes of this project and future projects in different settings and disease sites. Second, understanding the potential enablers and barriers to the use and sustainability of the synoptic MRI report will assist in the development of novel, successful, and cost-effective strategies to implement and sustain the use of the synoptic MRI report across centres.

## Discussion

This project will develop a synoptic MRI report for primary rectal cancer, and identify the enablers and barriers to the implementation and sustainability of this synoptic report in clinical practice. The synoptic MRI report created will be robust because it will be developed through an extensive literature review with rigorous qualitative research methods. Furthermore, the interviews with relevant stakeholders will elicit enablers and barriers to use and sustainability of synoptic reports in clinical practice and will be used to build upon a pre-existing framework of physician adherence [18]. In this way, a framework tailored specifically for clinical synoptic reports will be developed and used to develop novel, successful and cost-effective strategies for implementation of the synoptic MRI report, as well as other synoptic reports.

By improving the overall quality of MRI reporting, it is expected that improved communication between the members of the multidisciplinary care team will lead to better treatment decisions and ultimately lead to improved patient care and outcomes for rectal cancer patients in Ontario.

## Competing interests

The authors declare that they have no competing interests.

## Authors' contributions

EK, RM, AS, MF, LM, and AG have participated in the design of the study. AG and EK have expertise in qualitative research methods and will supervise data collection and analysis. All authors read and approved the final manuscript.

## Supplementary Material

Additional file 1 **Key criteria from preliminary literature review**. Results of a literature review on essential items for MRI report. Click here for file

Additional file 2 **Conceptual framework for physician adherence to new clinical interventions (taken from Cabana **[18]). Conceptual framework to describe the adoption of the synoptic report into practice. Click here for file
