# Supplementary material for: Development of a synoptic MRI report for primary rectal cancer
Source: Implement Sci. 2009 Dec 2;4:79. doi: 10.1186/1748-5908-4-79 (PMC3224933; doi:10.1186/1748-5908-4-79)
Supplement: Additional file 1 — Key criteria from preliminary literature review. Results of a literature review on essential items for MRI report. [file 1748-5908-4-79-S1.DOC]

|  | ***Item*** | ***Key Criteria*** | |
| --- | --- | --- | --- |
| 1 | Tumour Location | - upper rectum (10-15 cm) - mid rectum (5-10 cm) | - low rectum (0-5 cm)   - rectosigmoid |
| 2 | Tumour Size | - maximum cranio-caudal extent (tumour length) - maximum A-P diameter | - maximum tranverse diameter - circumferential extent of tumour using a clock face |
| 3 | Height of tumour from anal verge | - does not invade levator - distance from superior margin of anorectal junction to inferior aspect of tumour   - invades levator | - invades levators but does not extend into  intersphincteric space  - invades levators and invades the intersphincteric space  - invades levators and invade the external anal sphincter |
| 4 | T Stage | - T1:invades submucosa  - T2:invades muscularis propria  - T3: invades subserosa  - T3a : beyond muscularis propria < 1mm  - T3b : beyond muscularis propria 1-5 mm  - T3c : beyond muscularis propria >5-15 mm | - T3d : beyond muscularis propria >15 mm  - maximum depth of extramural spread beyond  muscularis propria in  mm  - T4 : invades adjacent organs  - T4a : invades adjacent organs  - T4b : perforation of peritoneal covering |
| 5 | Local Involvement | - Bladder - Ureter - Seminal vesicle - Prostate - Uterus | - Vagina - Sacrum - Levator Ani   - Pelvic side wall |
| 6 | Tumour Morphology | - Polypoid - Infiltrative | - Mucinous |
| 7 | Tumour Border | - Smooth - Nodular | - Infiltrating |
| 8 | Relation of tumour to circumferential margin | - shortest distance to mesorectal fascia - distance to mesorectal fascia < 1mm | - distance to mesorectal fascia > 1mm - location of shortest distance to mesorectal fascia (using clock face) |
| 9 | Mesorectal lymph nodes | - absent - present - suspicious - size | - location - mixed signal intensity or irregular border - tumour deposit |
| 10 | Extramural venous invasion | - no tumour signal in vessels - tumour signal intensity expanding small non-characterizable veins | - tumour signal intensity expanding to large anatomical veins (e.g. superior haemorrhoidal) - extramural venous invasion beyond mesorectal fascia |
|  | Other |  | |
