# Supplementary material for: Development of a synoptic MRI report for primary rectal cancer
Source: Implement Sci. 2009 Dec 2;4:79. doi: 10.1186/1748-5908-4-79 (PMC3224933; doi:10.1186/1748-5908-4-79)
Supplement: Additional file 2 — Conceptual framework for physician adherence to new clinical interventions (taken from Cabana [18]). Conceptual framework to describe the adoption of the synoptic report into practice. [file 1748-5908-4-79-S2.DOC]

**KNOWLEDGE**

**ACCEPTANCE/ATTITUDES**

**ADOPTION/ADHERENCE**

***Awareness/Familiarity***

- Volume of information
- Time needed to stay informed
- Accessibility
- Promotion
  - Opinion leader
  - Publications
  - Agency endorsement

***Agreement with:***

- Interpretation of evidence
- Ease of application/feasibility
  - “Too cookbook”
  - Too rigid to apply
- Challenge to autonomy
- Complexity (# of items)

***External/ Internal factors***

*External*

- Time constraints
- Lack of human resources
- Cost
- Organizational constraints
- Lack of reimbursement

*Internal*

- Age
- Sex
- Specialty
- Years in practice
- # cases/year

***Outcome expectancy***

- Belief that performance of task will lead to the desired outcome

***Current motivation***

- Habit
- Routine
- Incentives

***Self-efficacy***

- Ability to perform task
